# ﻿A note on the type of *Harpalyce* (Fabaceae, Brongniartieae), with description of two new local endemic species from Cuba

**DOI:** 10.3897/phytokeys.225.99321

**Published:** 2023-04-24

**Authors:** Rosa Gloria Rankin Rodríguez, Pedro Alejandro González Gutiérrez, Werner Greuter

**Affiliations:** 1 Jardín Botánico Nacional, Universidad de La Habana, La Habana, Cuba Universidad de La Habana La Habana Cuba; 2 Centro de Investigaciones y Servicios Ambientales de Holguín, Holguín, Cuba Centro de Investigaciones y Servicios Ambientales de Holguín Holguín Cuba; 3 Herbarium Greuter, Herbarium Mediterraneum Panormitanum, Orto Botanico, Università degli Studi, Via A. Lincoln 10, Palermo, Italy Università degli Studi Palermo Italy

**Keywords:** *Brongniartieae*, Eastern Cuba, *Fabaceae*, *Harpalyce*, leaf gland morphology, limestone substrate, serpentine, *Brongniartieae*, Cuba oriental, *Fabaceae*, *Harpalyce*, morfología de glándulas foliares, serpentinas, substratos calcáreos

## Abstract

Two new species of *Harpalyce* are described from Cuba, *H.revoluta***sp. nov.** from a serpentine area in the northern part of E. Cuba and *H.marianensis***sp. nov.** from calcareous areas in the southern part of E. Cuba. Both have relatively small flowers, with an up to 6 mm long standard, and 2–3 mm long wings. *Harpalycemarianensis* is further characterized by strongly suberous (corky) young branches of a spongy consistence, deeply furrowed longitudinally, and by leaflets covered with a particular type of orange, apparently disk-shaped, sessile glands abaxially. *Harpalycerevoluta*, moreover, has suborbicular or broadly elliptic leaflets with a strongly recurved or sometimes revolute margin, secondary veins inconspicuous on either side; the foliar glands, by their morphology and anatomy, are of a different type. An epitype is designated for the name *Harpalyce* and its type, *H.formosa*; the distribution of both new species and their close relatives is mapped, and an updated identification key is offered, to cater for all 16 currently recognised Cuban species.

## Abstract (es)

Se describen dos nuevas especies de *Harpalyce* para Cuba oriental, *H.revoluta***sp. nov.** de las áreas de serpentina del norte y *H.marianensis***sp. nov.** de áreas de formaciones calcáreas del sur. Ambas se caracterizan por tener flores pequeñas, con estandarte de hasta 6 mm de largo y alas de hasta 3 mm de largo. *Harpalycemarianensis* se distingue además por sus ramas jóvenes fuertemente suberosas, de consistencia esponjosa, profundamente surcadas longitudinalmente, y los folíolos cubiertos abaxialmente por un tipo particular de glándulas sésiles anaranjadas. *Harpalycerevoluta*, por su parte, tiene folíolos suborbiculares o anchamente ovales, con margen fuertemente recurvado o hasta revoluto, con venas secundarias inconspicuas en ambas caras, y glándulas foliares, por su morphología y anatomía, son de un tipo distinto. Se designa un epitipo para el nombre *Harpalyce* y su tipo, *H.formosa*. Se presenta un mapa de distribución de las nuevas especies y las relacionadas con ellas y se ofrece una clave de identificación actualizada, que incluye las 16 especies cubanas actualmente reconocidas.

## ﻿Introduction

*Harpalyce* DC. is one of those genera that, so to say, sprouts new species on a regular basis, with apparently increasing frequency. The phenomenon is not limited to Cuba, but can also be observed in the two other, disjunct areas in which the genus occurs: Central Mexico to Nicaragua in Central America, and large parts of Brazil (São-Mateus 2018; São-Mateus et al. 2019; Rankin and González 2021). In Cuba, it is due to a variety of factors, among which are the occurrence in small populations of only a few individuals, long overlooked by collectors; and the fact that many collected specimens are incomplete, often lacking flowers and fruits, and unsuited for identification below the species group level, so that undescribed species often remain unrecognised in the herbaria.

In Cuba, from where 16 species (including the two present, new ones) are now known, *Harpalyce* was first collected by Charles Wright in the 1860s. The species described by Grisebach (1866) based on Wright’s material remained the single Cuban one for well over half a century. In the 1920s, five more species were added, consequent to the intense collecting activities of the Swede E. L. Ekman and North American botanists based in New York. Cuban botanists recognised three more species, all described in 1950. Borhidi and Muñiz (1977) added no less than 5 species (not counting synonyms) based on the collections of local botanists kept in Cuban herbaria. Just recently, describing a new species of our own, we published an update of the genus for Cuba (Rankin and González 2021). Even then, we realised that our digest was by no means final, as several sterile specimens exist in Cuban herbaria, including the type material for species described by Borhidi and Muñiz (1977; see Rankin and González 2021) that cannot be placed reliably in the framework of the species so far described. Further field investigations are planned which, if successful, will yield complete, fertile material from the very same or nearby populations. Therefore, be warned: as soon as the so far incompletely known plants have been located and collected, further descriptions of new taxa will likely be forthcoming.

## ﻿Materials and methods

The original and complementary literature dealing with *Harpalyce* (Fabaceae, Brongniartieae) was consulted. In addition to the ca. 170 materials studied earlier (Rankin and González 2021), about 80 specimens (including duplicates) of Cuban *Harpalyce* belonging to the herbaria B, HAJB, JE, and PAL-Gr were examined, not counting the digital images of other specimens (including types) available online and housed in relevant herbaria such as A, GH, GOET, HAC, HAJB, K, MO, NY, P, S, UC, and US (see also Rankin and González 2021 and its Appendix 1 [https://doi.org/10.3372/wi.51.51204]). Herbarium codes follow Thiers (2022+). Georeferenced specimen label data were incorporated into the Flora Database of the Republic of Cuba (Greuter 2003); geographical coordinates were used to generate a distribution map, using the mapping software QIGS for Windows, version 3.16.

Leaf glands were studied using the stereo-zoom microscope system Olympus SZX16; the photographs were taken using the microscope’s camera Olympus DP72 and its CellSens Standard software. Cross cuts of the lamina were obtained by razor-cutting manually mature leaflets, previously soaked soap water at room temperature. Unstained cuts in aqueous solution were studied at 200× and 400× magnification by optical microscopy using a Carl Zeiss Axioscop instrument.

### ﻿Notes on the type of and author citation for the name *Harpalyce*

While searching the literature for potentially relevant information, we came across McVaugh’s (2000) book on the botany of Sessé and Mociño’s expedition to New Spain (i.e., Mexico). McVaugh’s excellent work includes a voluminous chapter on the scientific names of plants based on Sessé and Mociño’s collection of paintings that had been used by Candolle and others in describing new species, and sometimes genera, as in the case of *Harpalyce* and its type, *H.formosa*. The corresponding entry, on p. 316 of the book, gives details on what is probably the holotype of the species name, or, failing this, the lectotype designated by Arroyo (1976). It is an original painting, numbered 227, kept in the library of the Conservatoire botanique de la Ville de Genève (G), a copy of which is present, as #0607, in the Torner Collection of the Hunt Institute for Botanical Documentation; drawings of analytical details based on that painting can be viewed online (as https://fm-digital-assets.fieldmuseum.org/369/473/30332.jpg), being kept in the type photograph collection of the Botany Department, Field Museum of Natural History. McVaugh (l.c.) wrote that the depicted material was probably found in 1789 in the state Guerrero in South Mexico; he also shared Standley’s (1922) opinion that the type material is impossible to identify. No herbarium material of Sessé and Mociño corresponding to *H.formosa* is known to exist. Arroyo (1976) applied the name to a definite taxon, endemic to the south-eastern part of the state of Mexico and to the state of Puebla, but not known from Guerrero. We agree with her taxonomic assessment of the type illustration, but in view of the doubt expressed by Standley and McVaugh (ll. cc.), even with respect to generic placement, we believe it to be necessary to fix the application of the name by designating an epitype.

*Harpalyceformosa* DC., Prodr. 2: 523. 1825. Holotype [or, if lectotype, designated as such by Arroyo (1976: 40)]: Original painting (#227) of “*Astragalus Formosus*. Sp. N.” in the Candolle collection “Icones florae mexicanae” of the Sessé and Mociño paintings, and copies thereof, in the library of the Conservatoire botanique in Geneva (G). **Epitype (designated here)**: Tehuacán, Puebla, June 1905, *Purpus 1196* (UC 85301 [foto!]). This specimen was revised by Arroyo (1976) and considered by her as the holotype (more likely it is the lectotype) of *Harpalyceferruginea* Brandegee, a taxonomic synonym of *H.formosa*; it can be viewed on the Internet (https://global.plants.jstor).

A further point of note clarified by McVaugh (2000: 14–17) is the appropriate author citation for the generic name, which had often (e.g. by Arroyo 1976) been cited as “*Harpalyce* Mociño and Sessé ex de Candolle” but should, along with all similar cases of Candolle’s names based exclusively of the Icones Florae Mexicanae, be credited to DC. alone. This is abundantly clear in the case of *Harpalyce*, a genus that Mociño did not recognise, let alone propose a name for it, but that he included in *Astragalus*.

### ﻿Description of two new Cuban species of *Harpalyce*

#### ﻿ Harpalyce marianensis

Taxon classificationPlantaeFabalesFabaceae

R.Rankin, P.A.González & Greuter sp. nov.

DCDF6FEA-D1C4-50A4-A61C-3CD606771F09

urn:lsid:ipni.org:names:77318009-1

##### Type material.

***Holotype*. Cuba** – Guantánamo Prov. • San Antonio del Sur, Abra de Mariana en el barranco, 5 km al noroeste de San Antonio del Sur; [20.08828°N, 74.85846°W]; A. Álvarez de Zayas et al. HFC 43077; montes secos, caliza; 11 May 1980; JE 28983.

***Isotypes*. Cuba** • Same collection data as for preceding; B 100364878 [http://herbarium.bgbm.org/object/B100364878]; HAJB 1289, 1290; JE 28984.

##### Other material examined.

**Cuba** – **Guantánamo Prov.** • San Antonio del Sur, manigua costera cerca de Playa Baitiquirí; [20.02482°N, –74.85226°W]; May 1968, Bisse & Köhler HFC 9259; HAJB, JE; Baitiquirí, en el camino a la Mina del Yeso; [20.02482°N, 74.85226°W]; 11 Apr. 1972; Bisse & Berazaín HFC 21818; HAJB, JE; Abra de Mariana, loma al oeste del barranco; [20.08828°N, 74.85846°W]; 6 Feb. 1978; Bisse et al. HFC 36575; B 100362446, HAJB, JE; Abra de Mariana, loma al oeste del Abra; [20.08828°N, 74.85846°W]; 9 Feb. 1979; Berazaín et al. HFC 39129; B 100361112, HAJB, JE.

**Figure 1.**
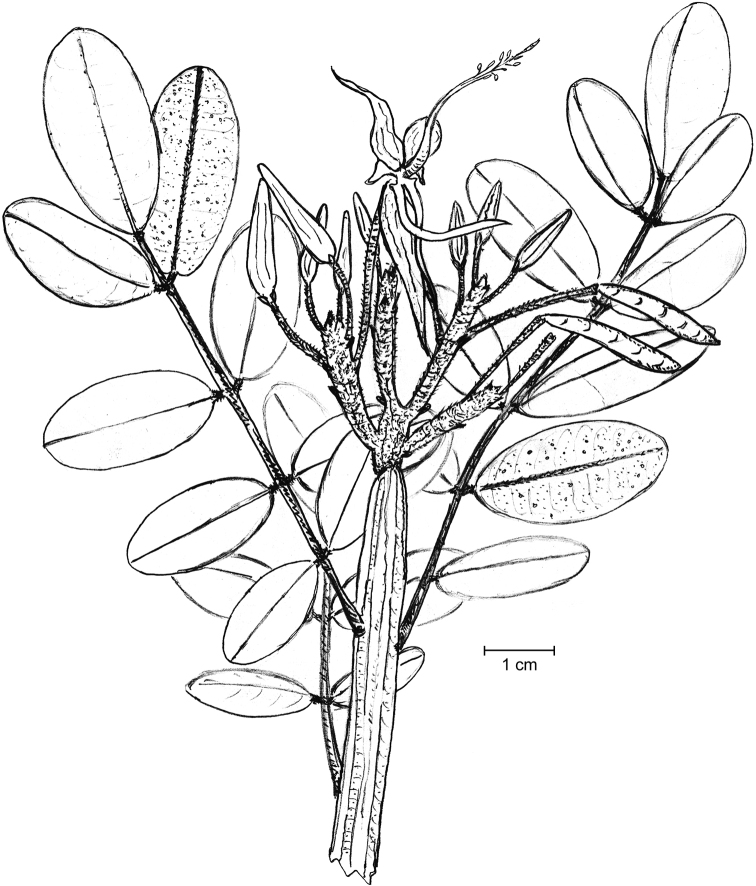
Flowering branch of *Harpalycemarianensis.* Drawing by PAGG from an isotype specimen (JE 28983).

**Figure 2.**
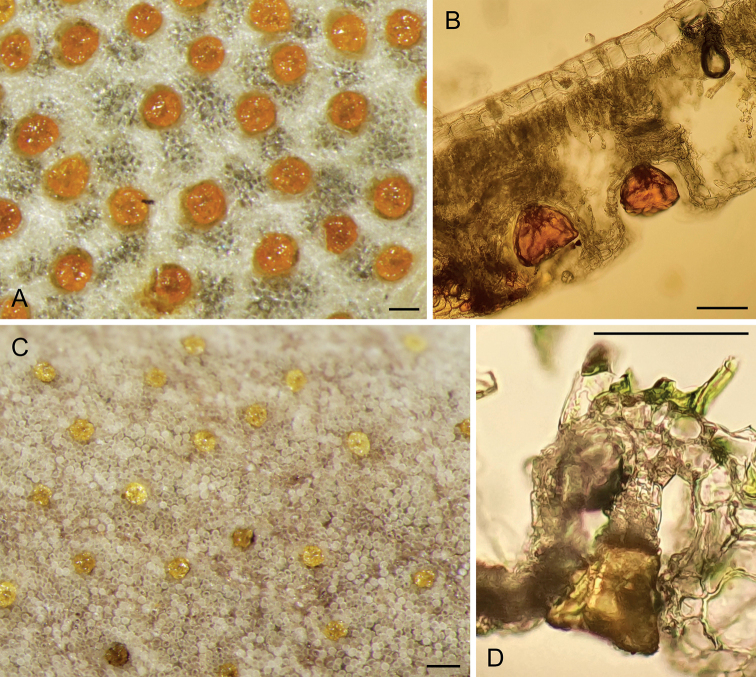
**A–D** leaf glands of *Harpalycemarianensis* (**A, B** from holotype specimen) and *H.revoluta* (**C, D** from specimen HFC 44956, B 100374694) **A, C** surface view **B, D** cross section. Preparations and photographs by Bibiana Moncada, Berlin. Scale bars: 100 μm.

**Figure 3.**
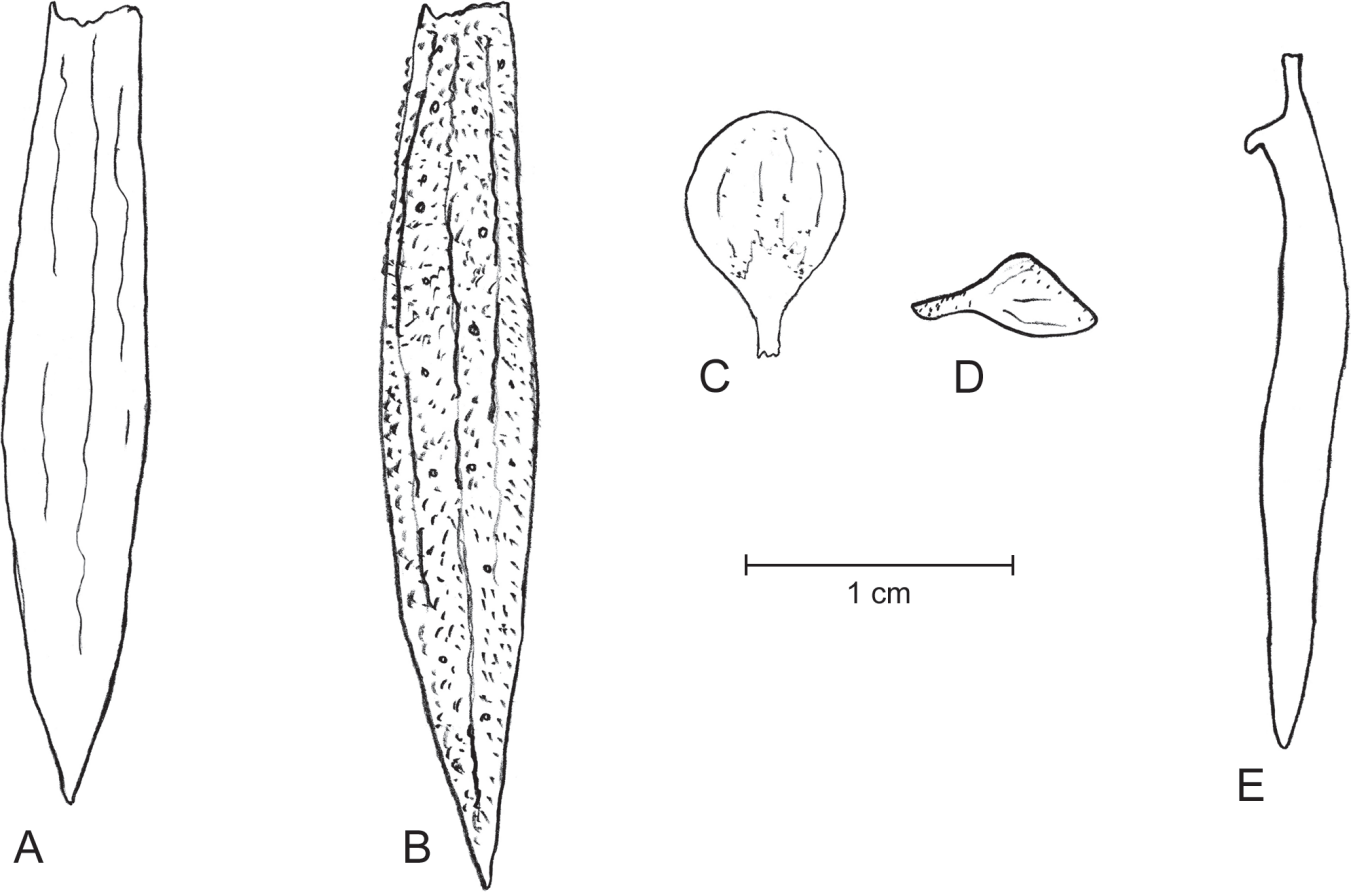
**A–E** analysis of the perianth of *Harpalycemarianensis***A** vexillary calyx lip, from inside **B** carinal calyx lip, from outside **C** standard, frontal view **D** wing **E** keel, lateral view. Drawings by PAGG from a flower of an isotype specimen (JE 28983).

Shrubs or small trees; young branches very suberous (corky), with a spongy appearance, deeply longitudinally ridged (Fig. 1), ferruginous-pubescent, old branches also suberous, but glabrescent. Stipules not seen, probably early shed. Leaves imparipinnate, normally alternate, or frequently subopposite, 4–7.5 cm long; petiole 10–18 mm long, densely ferruginous-pubescent; rachis 2–5.5 cm long, ferruginous-pubescent; leaflets (5-)7–9(-11) per leaf, coriaceous; lamina elliptic to narrowly elliptic, with a rounded base, the tip rounded or slightly emarginate, sometimes shortly mucronulate; the margin entire, mostly flat; adaxially glabrous, shiny (when dry), abaxially subglabrous except on the midvein; the leaflets, abaxially, are densely covered with showy orange, apparently disk-shaped glands of “type A” (the plump, apically flattened gland body is sessile in a wide open depression of the mesophyll: Fig. 2A, B); leaflet midvein sunk adaxially, prominent and ferruginous-pubescent abaxially, glabrescent with age, secondary veins in 6–9 pairs, ± conspicuous abaxially; lateral leaflets in opposite pairs, with a 1–1.5 mm long, thick, ferruginous-pubescent petiolule, the lamina 1.2–2.5(-3) × 0.8–1(-1.3) cm; terminal leaflet with a 1.5–2.5 mm long, thick and ferruginous-pubescent petiolule, the lamina 2–3.3 × 0.8–1.5 cm. Inflorescence terminal, many-flowered (with ≤ 50 flowers), 4–4.5 cm long, densely ferruginous-pubescent; bracts c. 1 mm long, densely ferruginous-pubescent; pedicels ≤ 2.5 cm long, ferruginous pubescent. Flowers zygomorphic. Calyx lips narrowly triangular, acute, outside densely covered with ferruginous hairs and sessile glands, inside glabrous or with scattered hairs; vexillary calyx lip (Fig. 3A) 3–3.2 × 0.5–0.6 cm, carinal calyx lip (Fig. 3B) 3.4–3.5 × 0.5–0.6 cm; petals membranaceous (when dry); standard (Fig. 3C) with a c. 2 mm long claw, the blade orbicular or broadly elliptic, apically rounded, 5–6 mm in diameter; wings (Fig. 3D) with a c. 2 mm long claw, the blade ± triangular, 2–3 × c. 2 mm, apparently lacking a basal auricle; keel petals (Fig. 3E) with a c. 2 mm long claw, the blades not connate, 2.3–2.5 × 0.2–0.3 cm, with a basal auricle on the vexillar side. Stamens 10; filaments 1.8–2.2 cm long, basally fused into a tube, distally free for 4–5 mm; anthers ± sagittate, c. 1.5 mm long (when dry). Pistil 3–3.2 cm long; ovary fusiform, laterally compressed, 7–8 × 1.5–2 mm, glabrous, 6–7-ovulate; style filiform, curved, 2.3–2.5 cm long, glabrous. Legume linear, 3–3.3 × c. 1 cm, brown, tipped with a c. 2 mm long mucro. Seeds up to 6 per legume, 4 × 1.5 mm (when dry).

##### Phenology.

Collected in flower and with fruits in April and May.

##### Etymology.

Named after the type locality, Abra de Mariana.

##### Distribution.

**Figure 4.**
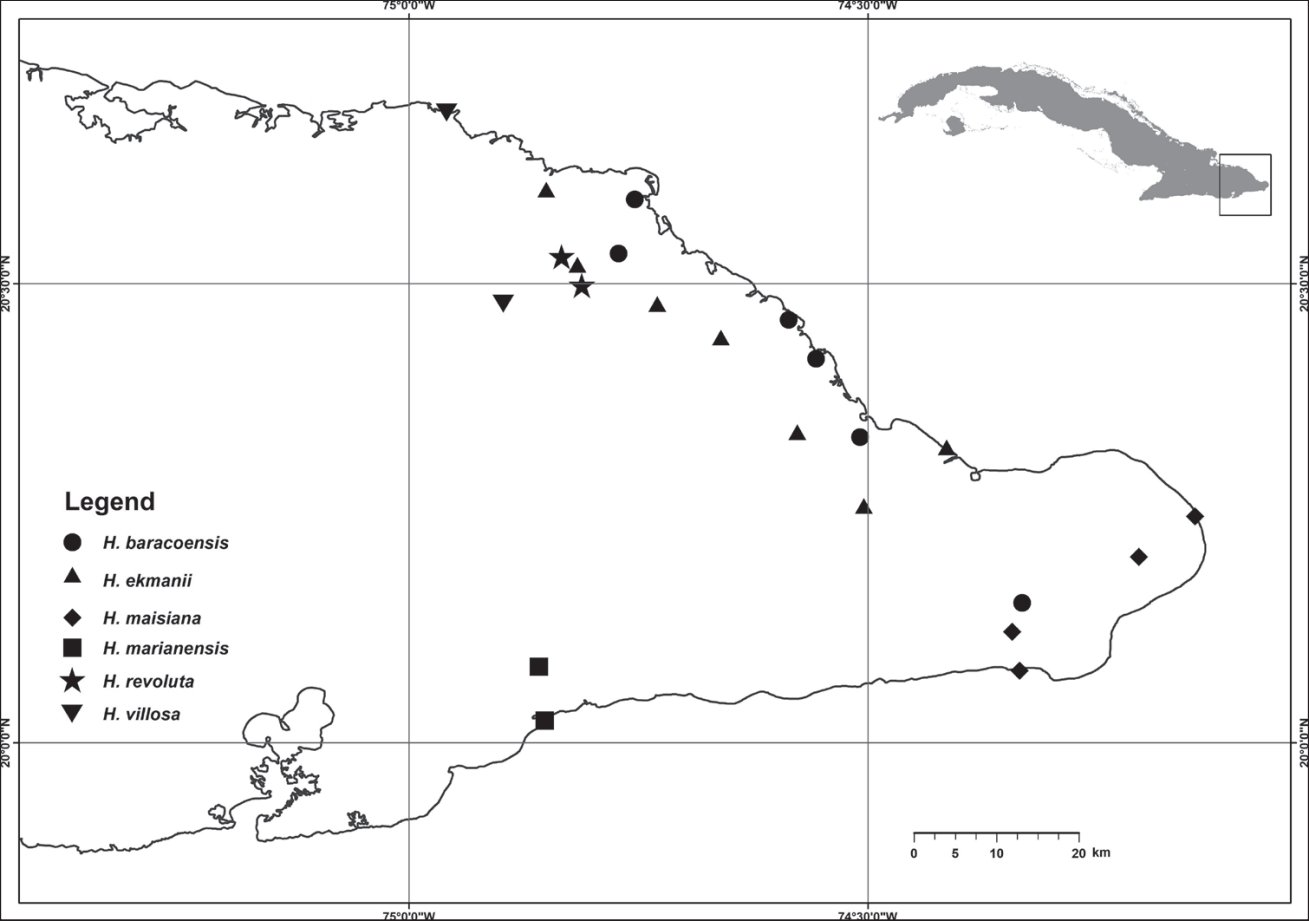
Distribution of the two new species and of related, small-flowered *Harpalyce* species with free keel petals endemic to E Cuba.

Southern part of E Cuba, province Guantánamo, municipality San Antonio del Sur: Abra de Mariana and Baitiquirí. Grows in dry scrub vegetation on limestone substrate. Fig. 4.

##### Affinities and diagnostic features.

*Harpalycemarianensis* has morphological affinities with *H.revoluta* (see below) and other Cuban species that have a relatively small (≤ 8–9 mm in length or diameter), orbicular to broadly elliptic standard and free keel petals, such as: *Harpalyceekmanii* Urb., *H.villosa* Britton & P. Wilson, and *H.baracoensis* Borhidi & O.Muñiz, particularly with *H.ekmanii*, with which it also shares glabrous or subglabrous leaflets; however, *H.ekmanii* has larger leaflets (2.5–7.5 × 1.5–3 cm vs 1.2–3.3 × 0.8–1.5 cm). *Harpalycemarianensis* has pronounced suberous young branches with a spongy consistence and deep longitudinal ridges, a feature that is not found in *H.ekmanii*. Also, *H.marianensis* grows in dry scrub on limestone in the southern part of E Cuba, whereas *H.ekmanii* grows in rainforest, pine woods or dry scrub on ultramaphic soils such as serpentines in the northern portion of E Cuba.

Sterile plants or specimens of *Harpalycemarianensis* might be confused with *H.maisiana* León & Alain, a species that also occurs in limestone areas of southern E. Cuba, but these two can be distinguished from each other through the colour of the glands on the adaxial face of the leaflets, yellow in *H.maisiana* vs orange in *H.marianensis*. When in flower, they can be easily told apart: the standard is oblanceolate, 1 × 0.2–0.3 cm and apically slightly emarginate in *H.maisiana* but orbicular or broadly elliptic, 5–6 mm in diameter or length and apically rounded in *H.marianensis*; the keel petals are partially connate in the apical third and 1.2–1.3 × 0.2–0.3 cm in *H.maisiana*, but totally free and 2.3–2.5 × 0.2–0.3 cm in *H.marianensis*.

#### ﻿ Harpalyce revoluta

Taxon classificationPlantaeFabalesFabaceae

P.A.González, R.Rankin & Greuter sp. nov.

FA837DDF-E3D0-5DE9-B5C7-D940F7FA34B0

urn:lsid:ipni.org:names:77318010-1

##### Type material.

***Holotype*. Cuba – Holguín Prov.** • Cuchillas de Moa; alrededores del aserrío La Melba; A. Álvarez de Zayas et al. HFC 42194; charrascales altos con *Bonnetiacubensis*; [20.51944°N, 74.81778°W]; alt.450–500 m; 28 Apr. 1980; B 100364586 [http://herbarium.bgbm.org/object/B100364586].

***Isotype*s. Cuba** • Same collection data as for preceding; HAJB 1286-1288, JE 28985.

##### Other material examined.

**Cuba** – **Holguín Prov.** • Moa; La Melba; charrascal cerca del aserrío; [20.51944°N, 74.81778°W]; Mar. 1968; Bisse & Köhler HFC 7066; HAJB, JE; La Melba, charrascal cerca del aserrío; [20.51944°N, 74.81778°W]; Mar. 1968; Bisse & Köhler HFC 7626; HAJB, JE; La Melba; charrascal cerca del aserrío; [20.51944°N, 74.81778°W]; 22 Dec. 1968; Bisse & Lippold HFC 11062, 11329; HAJB, JE; Cayo Probado; orillas de las cabezadas del río Jiguaní; [20.48864°N, 74.82188°W]; 3 Apr. 1972; Bisse & Berazaín HFC 21968; B 100462873, HAJB, JE; alrededores del aserrío La Melba; [20.51944°N, 74.81778°W]; 25 Apr. 1981; Bisse et al. HFC 44956; B 100374694, HAJB, JE.

**Figure 5.**
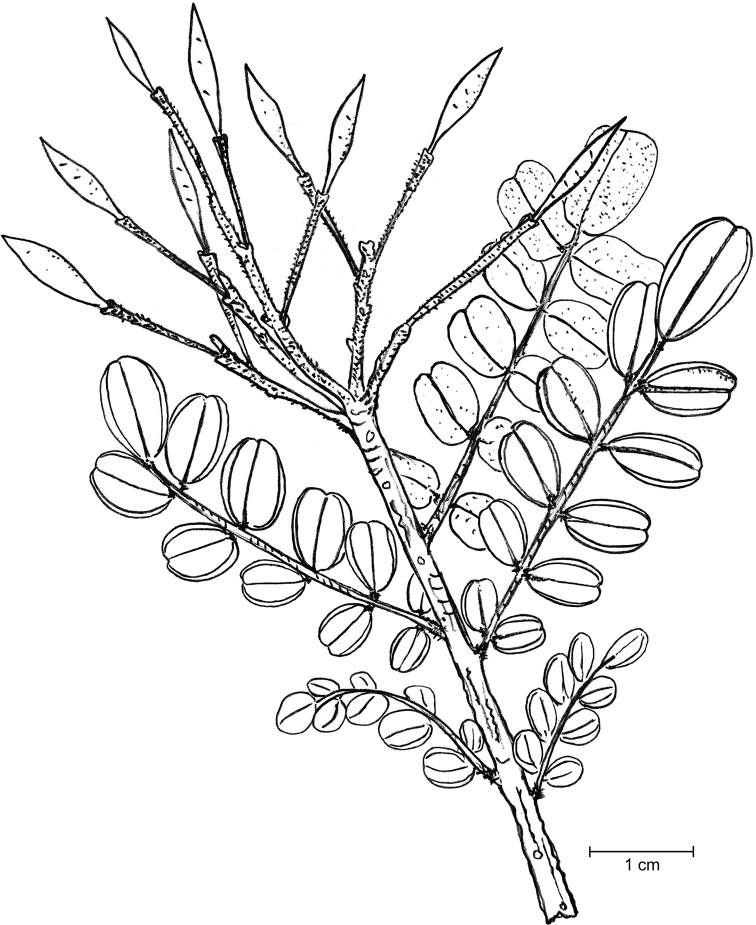
Branch with immature fruits of *Harpalycemarianensis.* Drawing by PAGG from the holotype specimen.

**Figure 6.**
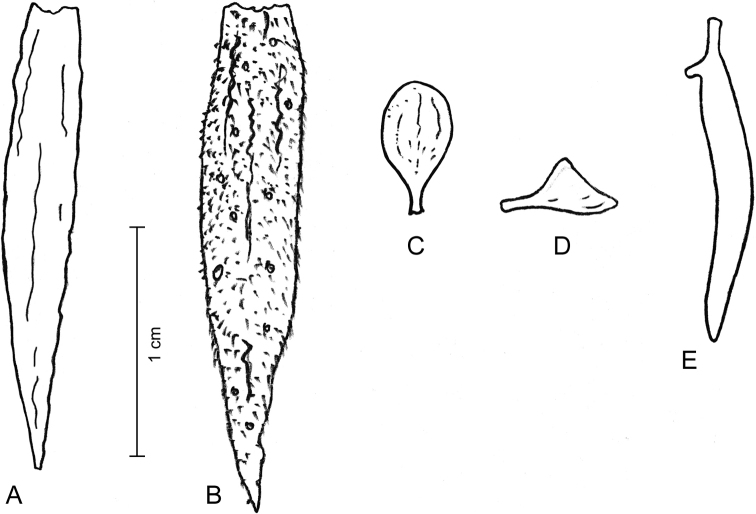
**A–E** analysis of the perianth of *Harpalycerevoluta***A** vexillary calyx lip, from inside **B** carinal calyx lip, from outside **C** standard, frontal view **D** wing **E** keel, lateral view. Drawings by PAGG from a flower of specimen HFC 44956, B 100374694.

Shrubs or small trees; young branches pubescent, old branches (Fig. 5) suberous and glabrescent. Stipules not seen, probably early shed. Leaves imparipinnate, normally alternate, sometimes subopposite, 3–7 cm long; petiole 0.5–10 mm long, very thick particularly at the basis, pubescent; rachis 2–6 cm long, pubescent, glabrescent with age; leaflets 7–11 per leaf, rigidly coriaceous, opposite or very rarely subopposite; lamina mostly suborbicular or ± broadly elliptic, with a rounded or obtuse base, the tip emarginate; margin entire, recurved to strongly revolute; the surface shiny (when dry), adaxially glabrous, abaxially glabrous except on the midvein and beset with well-spaced yellow to orange, somewhat funnel-shaped glands of “type B” (in which the obconical gland body is stalked in the bottom of a mesophyll cavity, barely protruding through its narrowed opening; Fig. 2C, D); leaflet midvein sunk adaxially, prominent and with some scattered hairs abaxially, secondary veins inconspicuous on both faces; lateral leaflets with a c. 1 mm long, thick and ferruginous-pubescent petiolule, the lamina 0.6–1.2 × 0.5–1 cm, terminal leaflets with petiolule 1–3 mm long, thick and ferruginous pubescent, lamina 1–2 × 0.8–1 cm. Inflorescence terminal, with ≤ ca. 20 flowers, 2–4 cm long, densely ferruginous-pubescent; peduncle 1–1.5 cm long, densely ferruginous-pubescent, bracts linear, 2.5–3 mm long, densely ferruginous-pubescent; pedicels 1–1.5 cm long, densely ferruginous-pubescent. Flowers (Fig. 5) zygomorphic. Calyx lips narrowly triangular, acute, outside densely covered with ferruginous hairs and sessile glands, inside glabrous and with scattered glands; vexillary calyx lip (Fig. 6A) 2.1 × 0.3 cm, carinal calyx lip (Fig. 6B) 2.3 × 0.4 cm; petals membranaceous (when dry); standard (Fig. 6C) with a 2–2.5 mm long claw, the blade broadly elliptic, apically rounded, c. 3.5 × 2.5 mm; wings (Fig. 6D) with a c. 2 mm long claw, the blade ± triangular, c. 2 × 2 mm, apparently lacking a basal auricle; keel petals (Fig. 6E) with a 2–3 mm long claw, the blades not connate, 1.2–1.3 × c. 0.2 cm, with an auricle on the vexillar side. Stamens 10; filaments 1.7–2 cm long, basally fused into a tube, distally free for 4–5 mm; anthers sagittate, c. 1.5 × 0.7 mm (when dry). Pistil c. 2.3 cm long; ovary fusiform, laterally compressed, 4 × 1–1.5 mm, glabrous; style filiform, 1.9–2 cm long, glabrous. Legume (immature) linear, 13–14 × 3–3.5 mm. Seeds not seen.

##### Phenology.

When collected in late April, the type material was in flower and with immature fruits. Some other specimens seen, collected in April as well, similarly were in flower and with immature fruits.

##### Distribution.

Northern part of E Cuba, province Holguín. Grows in rainforest, pine woods or xeromorphic scrub on ultramaphic substrate (serpentine). Fig. 4.

##### Etymology.

The epithet refers to the characteristic, revolute leaflet margin.

##### Affinities and diagnostic features.

*Harpalycerevoluta*, shows morphological affinities with *H.marianensis* (see above) and other Cuban species that have a relatively small (≤ 8–9 mm in length or diameter), orbicular to broadly elliptic standard and free keel petals, particularly with *H.ekmanii*, with which it shares glabrous or subglabrous leaflets; however, in *H.ekmanii* the leaflets are much larger (2.5–7.5 × 1.5–3 cm vs 0.6–2 × 0.5–1 cm), with flat (vs recurved to revolute) margins, and the sessile glands on the abaxial leaf face are fairly dense, less spaced. The size and shape of the standard relates *H.revoluta* to *H.villosa*, but the latter has leaflets that, particularly when young, are densely hairy abaxially, not glabrous except on the loosely hairy midvein, as in *H.revoluta*.

### ﻿An update of the identification key for Cuban *Harpalyce*

In their recent survey of Cuban *Harpalyce*, Rankin and González (2021) presented a key for identifying the species they recognise. To account for the present, additional species, that key needs some modification. That key is reproduced here, with its 8^th^ dichotomy changed and expanded to account for the new species.

**Table:** 

1	Leaflets (especially when young) densely hairy, tomentose or pubescent abaxially, indumentum obscuring glands until leaflets are older and hairs are less dense	**2**
–	Leaflets glabrous, subglabrous or with scattered hairs, these never obscuring glands	**7**
2	Bracts leaf-like (foliose), 1–1.5 cm long	***Harpalycetoaensis***
–	Bracts small, linear or triangular, < 3 mm long	**3**
3	Leaflets (on dried specimens) green or greyish green adaxially, abaxially covered with a brownish yellow tomentum	**4**
–	Leaflets (on dried specimens) brown or dark grey adaxially, abaxially pubescent or puberulous, with brown or reddish brown hairs	**5**
4	Leaflet margin recurved or flat, or in some leaflets revolute; inflorescence 1–6-flowered	***Harpalycenipensis***
–	Leaflet margin consistently revolute; inflorescence always 1-flowered	***Harpalyceacunae***
5	Petiolule of terminal leaflet 1–3 mm long	***Harpalycevillosa***
–	Petiolule of terminal leaflet 4–10 mm long	**6**
6	Leaflets 5–11 per leaf; secondary veins conspicuous abaxially	***Harpalycealainii***
–	Leaflets 11–17 per leaf; secondary veins inconspicuous abaxially	***Harpalycebaracoensis***
7	Keel petals completely free from each other	**8**
–	Keel petals connate for most of their length or at least in apical third	**11**
8	Standard 1 cm in diameter; wings c. 1.5 cm long	***Harpalycecristalensis***
–	Standard 0.35–0.9 cm in diameter or length; wings 0.2–0.7 cm long	**9**
9	Leaflets mostly suborbicular or ± broadly elliptic, their margin recurved to strongly revolute; secondary veins inconspicuous on both faces	***Harpalycerevoluta***
–	Leaflets elliptic to narrowly elliptic, oblong or oblong-elliptic, their margin flat or almost so; secondary veins commonly conspicuous, at least abaxially	**10**
10	Young branches very suberous, with a spongy consistence, deeply longitudinally ridged; leaflets 1.2–3.3 × 0.8–1.5 cm	***Harpalycemarianensis***
–	Young branches without a spongy appearance, not longitudinally ridged; leaflets 2.5–7.5 × 1.5–3 cm	***Harpalyceekmanii***
11	Standard ≤ 1.5 cm long	**12**
–	Standard ≥ 2 cm long	**13**
12	Petioles 1.5–2 cm long; standard c.1.5 × 0.8–1 cm; wings c. 1.5 cm long, keel petals c. 1.7 cm long	***Harpalyceborhidii***
–	Petioles 0.6–1.2 cm long; standard c. 1 × 0.2–0.3 cm; wings 1–1.1 cm long, keel petals 1.2–1.3 cm long	***Harpalycemaisiana***
13	Leaflets subopposite or alternate; wings black toward base, keel petals mostly black, with a yellow margin; legume 4–5 cm long	***Harpalycegreuteri***
–	Leaflets opposite; wings and keel petals red, orange, yellow or white; legume either up to 2.3 cm or at least 5 cm long	**14**
14	Legume 5–6.5 cm long	***Harpalycemacrocarpa***
–	Legume 1–2.3 cm long	**15**
15	Leaflets abaxially with well-spaced glands, these not touching each other	***Harpalycecubensis***
–	Leaflets abaxially with dense, partly contiguous or confluent glands	***Harpalycesuberosa***

## ﻿Discussion

As we stated in the introduction, the two new species here described are likely not the last addition to the genus. There are incomplete specimens in the Cuban herbaria, HAC and HAJB in particular, that in all likelihood represent new, undescribed species, which we shall duly name as soon as we succeed in relocating them in the field and collect complete, fertile material; furthermore, in view of the species definition we here adopt, following the tradition established by other authors, any newly discovered *Harpalyce* population is likely to represent a new taxon.

The question may be asked legitimately: is such a narrow species definition practical and defendable? We ignore whether our species are valid under a biological species concept. For all we know, they might all, or at least some of them, be freely interbreeding when brought together. Without much experimental work involving artificial crossing, it is impossible to know whether and to which extent evolutionary divergence has succeeded in establishing genetic barriers and cross-incompatibility between morphologically distinct populations. We are similarly ignorant of the extent of genetic isolation, if any, for many and indeed most species currently recognised in polymorphic Cuban genera. It is therefore legitimate to recognise morphologically distinguishable populations as separate taxa, and currently, no workable alternative to this course is available.

The role of small, isolated populations in evolution and speciation in higher plants has been studied by Runemark in the Aegean archipelago of Greece. He has coined the expression “reproductive drift” (Runemark 1969, 1970) to describe and explain this phenomenon. His ideas have not received the attention that they deserve, and we would like to encourage others to apply them to understand and explain the polymorphism observed in Cuban species complexes with a comparable fragmented population structure which, in the case of Cuba, is usually associated with edaphic specialisation to small and scattered habitats which, for biological purposes, are comparable to archipelagos of small islands in a sea of generalistic environmental conditions.

Concerning the general evolutionary context in which the Cuban populations are to be seen, it is useful to be mindful of the results and conclusions of recent phylogenetic studies (São Mateus 2018). That work was based on sequence analyses of both nuclear ribosomal and plastid DNA of an impressive sampling of taxa from Brasil (Harpalycesect.Brasilienses Arroyo) and Mexico (H.sect.Harpalyce) but only a single one from Cuba (two accessions of, allegedly, *H.cubensis* Griseb., which in fact, however, both belong to *H.suberosa* Urb.) representing H.sect.cubenses Rydb.). By São Mateus’ (2018) results, the genus *Harpalyce* and, presumably, its three geographically vicariant sections are natural, monophyletic taxa, with H.sect.Brasilienses being the sister clade of the two remaining sections, which are in turn sisters to each other (a rather shaky assumption, as long as a single Cuban taxon has been studied). The genus is old, assumed to be of Oligocene origin (> 30 Ma b.p.). Divergence of the northern sections from the Brazilian clade is also old (> 20 Ma b.p.). Species diversification, however, is fairly young, basically post-Miocene, an assumption that obviously, for Cuba, remains to be verified by the study of a larger number of taxa.

A further promising field of additional studies that we propose to follow up in the near future has opened up through the preliminary study of leaf glands of the new species here described. We found that the slight but obvious difference of the gland pattern they presented was based on obvious structural differences, to be observed on leaf transects, which have led to the definition of two distinct gland types that we have named “type A” and “type B”. Screening the remaining species of the genus for the presence in them of these gland types, and of possible further types yet to be defined, is a promising field of study. Preliminary results of leaf surface studies indicate that the “type A” glands of *Harpalycemarianensis* are also found in *H.ekmanii*, *H.maisiana* as well as *H.greuteri* R.Rankin & P.A.González (not discussed here); whereas “type B” glands of *H.revoluta* occur in *H.cristalensis* Borhidi & O.Muñiz. The scattered glands of the leaves of *H.villosa*, *H.baracoensis*, and *H.acunae* Borhidi & O.Muñiz, hidden by dense indumentum, might represent yet another type.

## Supplementary Material

XML Treatment for Harpalyce marianensis

XML Treatment for Harpalyce revoluta
